# Fluvoxamine Mediates Specific, Early, and Delayed SARS-CoV-2 Protection through Antioxidant and Cytoprotective Pathways via Sigma-1 Receptor Agonism

**DOI:** 10.34172/apb.2023.045

**Published:** 2022-09-18

**Authors:** Konstantinos Papadopoulos, Alexandra Papadopoulou, Tar Choon Aw

**Affiliations:** ^1^THAI StemLife, 566/3 Soi Ramkhamhaeng 39 (Thepleela 1), Prachaouthit Rd., Wangthonglang, Wangthonglang, Bangkok 10310, Thailand.; ^2^Occupational and Environmental Health Services, Feelgood Lund, Ideon Science Park, Scheelevägen 17, 223 63 Lund, Sweden.; ^3^Department of Laboratory Medicine, Changi General Hospital, 2 Simei Street 3, Singapore 529889.

## To Editor,

 Fluvoxamine is an affordable and widely available selective serotonin reuptake inhibitor (SSRI) antidepressant which is associated with reduced hospitalization, reduced severity, and mortality in outpatients with coronavirus disease 2019 (COVID-19).^1,2^ Fluvoxamine’s mechanisms of action in COVID-19 have been extensively discussed and is mainly mediated via its potent (Ki = 36 nM) sigma-1 receptor (sigma-1R, σ1R, S1R) agonism.^3-5^ Sigma-1R interferes with the early steps of virus-induced host cell reprogramming and protects against mitochondrial damage and endoplasmic reticulum (ER) stress in response to severe acute respiratory syndrome coronavirus-2 (SARS-CoV-2) infection.^4^ The reduction of cytokine production as indicated by sigma-1R’s key role in systemic inflammation is supported by substantial evidence.^4^ Finally, additional mechanisms may include acid sphingomyelinase (ASM) inhibition.^4^ SARS-CoV-2 activates host ASM leading to ceramide accumulation-a product of ASM catalyzed sphingomyelin-which aids in SARS-CoV-2 cell entry. Unlike fluvoxamine, chlorpromazine-another ASM inhibitor-did not lead to reduced mortality in patients with COVID-19 implying that ASM inhibition may not be clinically relevant.^6^

 We wish to highlight additional sigma-1R pathways that may be more pertinent in engendering fluvoxamine’s specific, early, and delayed SARS-CoV-2 protection. Sigma-1R agonists fluvoxamine and dehydroepiandrosterone (DHEA) have been shown-in both *in vivo* and *in vitro* systems-to mediate robust cardioprotection through increases in protein kinase B (Akt) phosphorylation, endothelial nitric oxide (NO) synthase (eNOS) levels and eNOS phosphorylation.^7,8^ In addition, fluvoxamine’s cardioprotective effect is mediated via upregulation of cardiac sigma-1R expression.^7^ Furthermore, stimulation of central nervous system sigma-1R may also inhibit sympathetic hyperactivation along with direct fluvoxamine stimulation of cardiac sigma-1R synergistically to improve hypertrophic cardiac dysfunction.^7^ Newer studies also suggest that fluvoxamine stimulation of sigma-1R reduces susceptibility to right ventricular dysfunction, atrial fibrillation, and ventricular arrhythmias and enhances cardiac function by upregulating sigma-1R protein content, mitigating myocardial fibrosis, sympathetic and electrical remodeling.^9^ Treatment with sigma-1R antagonists negated fluvoxamine-mediated eNOS upregulation and Akt-mediated eNOS phosphorylation. Moreover, all fluvoxamine-mediated cardioprotective effects are reduced, confirming that the sigma-1R modulates eNOS activity in the heart and blood vessels.^7,9^ Pertinent to COVID-19-related lung afflictions, sigma-1R agonism alleviates airway inflammation and airway remodeling via increased expression of AMP-activated protein kinase (AMPK) and inhibition of C-X-C chemokine receptor type 4 (CXCR4) expression while sigma-1R, AMPK, and CXCR4 antagonisms reversed these protective effects.^10^ Increased AMPK signaling appears beneficial in lung pathology as it reduces inflammatory responses in lung emphysema, mitigates pulmonary hypertension, and protects against lipopolysaccharide-induced acute lung injury and acute respiratory distress syndrome (ARDS).^11^ AMPK, a master cellular energy and redox-sensing protein, activated through several physiological and pathological conditions-such as hypoxia, caloric restriction, physiological exercise, aspirin and metformin-is also known to robustly induce eNOS in the endothelium.^12^

 Sigma-1R’s direct and AMPK-mediated-indirect effects on eNOS levels and phosphorylation, synergistically increase NO-production and bioavailability that underlie fluvoxamine’s and DHEA’s cardio- and vasculo-protective actions and mechanistically support specific and immediate anti-SARS-CoV-2 effects.^3,5^ Increased NO-generation and bioavailability may counteract SARS-CoV-2-induced endotheliitis and even inhibit SARS-CoV-2 replication and infection at an early stage as shown by the inhibition of (1) the palmitoylation of the SARS-CoV-1/2 spike protein, essential for fusion to the angiotensin converting enzyme (ACE)2, its obligate receptor, and (2) early production of viral RNA by inhibiting SARS-CoV-2 main protease. Both these processes are critical for membrane fusion and virion infectivity.^13^ In addition, inhibition of acetyl-CoA carboxylase by AMPK will directly inhibit palmitate synthesis further engendering anti-SARS-CoV-2 effects.^14^ Later in the course of SARS-CoV-2 infection, sigma-1R/AMPK/eNOS-induced increase in NO-production and bioavailability may promote delayed cardiopulmonary, renal and vascular protection through lower oxidative stress, apoptosis, and reduced systemic inflammatory responses.^15^ In severe COVID-19 and related ARDS, lower soluble eNOS levels have been reported implying that pharmaceutical interventions that increase NO-generation and bioavailability, may protect patients from severe lung complications.^16^

 Finally, sigma-1R agonism significantly increases nuclear factor erythroid-derived 2-like 2 (Nrf2), a master regulator of inducible antioxidant responses, and heme oxygenase-1 (HO-1) expression in astrocytes. Thus, Nrf2 may have therapeutic potential in neuroinflammation and protection against oxidative stress with implications for long COVID-19 neurological complications.^17^ Sigma-1R induction of AMPK will also phosphorylate Nrf2 resulting in subsequent expression of antioxidant genes.^12^ Interestingly, expression of Nrf2-dependent genes is suppressed in biopsies from COVID-19 patients.^18^ Recent data suggest that SARS-CoV-2 indeed represses the Nrf2/HO-1 antioxidant pathway while treatment of cells with Nrf2 agonists induced a strong antiviral program that limits SARS-CoV-2 replication.^18^

 In conclusion, sigma-1R agonism promotes specific eNOS/NO-, AMPK-, and Nrf2/HO-1-mediated defense mechanisms that actively suppress SARS-CoV-2 replication and transmission along with robust cardiovascular and neuroprotective effects. We thus strongly support the use of fluvoxamine as an affordable and widely available sigma-1R agonist for specific, early, and delayed protection against SARS-CoV-2 infection.

## Competing Interests

 The authors declare that they have no known competing financial interests or personal relationships that could have appeared to influence the work reported in this paper.

## Ethical Approval

 Not applicable.

## Funding

 This research did not receive any specific grant from funding agencies in the public, commercial, or not-for-profit sectors.
